# Ability of a selfish B chromosome to evade genome elimination in the jewel wasp, *Nasonia vitripennis*

**DOI:** 10.1038/s41437-023-00639-0

**Published:** 2023-07-31

**Authors:** Haena Lee, Pooreum Seo, Salina Teklay, Emily Yuguchi, Elena Dalla Benetta, John H. Werren, Patrick M. Ferree

**Affiliations:** 1 W. M. Keck Science Department, Claremont McKenna, Pitzer and Scripps Colleges, Claremont, CA 91711 USA; 2 Division of Biological Sciences, Section of Cell and Developmental Biology, University of California, San Diego, La Jolla, CA 92093 USA; 3 Department of Biology, University of Rochester, Rochester, NY 14627 USA

**Keywords:** Cytogenetics, Development

## Abstract

B chromosomes are non-essential, extra chromosomes that can exhibit transmission-enhancing behaviors, including meiotic drive, mitotic drive, and induction of genome elimination, in plants and animals. A fundamental but poorly understood question is what characteristics allow B chromosomes to exhibit these extraordinary behaviors. The jewel wasp, *Nasonia vitripennis*, harbors a heterochromatic, paternally transmitted B chromosome known as paternal sex ratio (PSR), which causes complete elimination of the sperm-contributed half of the genome during the first mitotic division of fertilized embryos. This genome elimination event may result from specific, previously observed alterations of the paternal chromatin. Due to the haplo-diploid reproduction of the wasp, genome elimination by PSR causes female-destined embryos to develop as haploid males that transmit PSR. PSR does not undergo self-elimination despite its presence with the paternal chromatin until the elimination event. Here we performed fluorescence microscopic analyses aimed at understanding this unexplained property. Our results show that PSR, like the rest of the genome, participates in the histone-to-protamine transition, arguing that PSR does not avoid this transition to escape self-elimination. In addition, PSR partially escapes the chromatin-altering activity of the intracellular bacterium, *Wolbachia*, demonstrating that this ability to evade chromatin alteration is not limited to PSR’s own activity. Finally, we observed that the rDNA locus and other unidentified heterochromatic regions of the wasp’s genome also seem to evade chromatin disruption by PSR, suggesting that PSR’s genome-eliminating activity does not affect heterochromatin. Thus, PSR may target an aspect of euchromatin to cause genome elimination.

## Introduction

Numerous plant and animal genomes contain extra, or supernumerary, chromosomes called B chromosomes, which are non-essential for the organism. B chromosomes are intriguing because they exhibit exceptional behaviors including mitotic drive, meiotic drive, and elimination of the essential, or A, chromosomes (Jones et al. 2008; Jones 2018; Houben 2017). These behaviors, generally referred to as B drive, can lead to an over-representation of B chromosomes in the progeny of B-carrying individuals (Houben 2017), thereby offsetting the tendency of B chromosomes to be lost because they are not needed. Of particular interest is understanding what unique sequence- and chromatin-level characteristics allow B chromosomes to drive, thereby distinguishing B chromosomes from the A chromosomes.

**Fig. 1:**
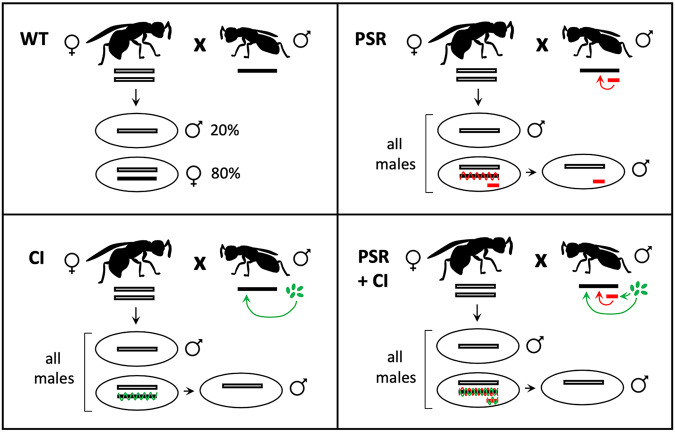
PSR and *Wolbachia* alter the sperm-inherited chromatin to cause PGE and a male-biased sex ratio. Top left: in a wild-type cross, approximately 20% of eggs are unfertilized, and they develop into haploid males. The other 80% of eggs are fertilized; these eggs, containing a chromosome set from each parent, develop as diploids into females. Top right: PSR (small red bar) is transmitted from the father into fertilized eggs. The activity of PSR (red arrow) alters the paternal chromatin, and this alteration (red wavy line) causes paternal genome elimination (PGE). This action converts diploid eggs into haploids, which develop into PSR-carrying, transmitting males. Bottom left: *Wolbachia* (green ovals) alters the paternal chromatin during sperm formation (green arrow). The altered chromatin (green wavy line) undergoes PGE, leading to haploid male development. Bottom right: in males carrying both PSR and *Wolbachia*, each agent alters the paternal chromatin, resulting in PGE. *Wolbachia* also modifies PSR, partially blocking its ability to segregate. In the latter three scenarios, PGE results in all-male broods.

One of the most striking examples of B drive stems from a B chromosome known as paternal sex ratio (PSR), which is found in certain populations of the jewel wasp, *Nasonia vitripennis* (Nur et al. 1988). PSR, a heterochromatic chromosome about a fourth the size of the smallest essential wasp chromosome, is transmitted strictly from father to progeny via the sperm (i.e., paternally) (Werren 1991; Werren and Stouthamer 2003). When present, PSR somehow alters the chromatin state of the sperm-derived half of the genome so that it fails to resolve into individualized chromosomes during its entry into the first embryonic mitotic division (Werren et al. 1987; Nur et al. 1988). This effect causes the sperm-derived chromatin to form a paternal chromatin mass (PCM). The PCM is incapable of segregating into daughter nuclei and is eventually lost during the subsequent mitotic cleavage divisions. Because PSR has no such effect on the egg-derived chromatin, and the sperm- and egg-derived sets are physically separated during this first division, the egg-derived chromatin successfully resolves into chromosomes and segregates, forming haploid daughter nuclei. The jewel wasp, like all hymenopteran insects, reproduces through haplo-diploidy, in which males develop as haploids from unfertilized eggs while females arise as diploids from fertilized eggs (Whiting 1967). The embryos stemming from eggs fertilized by PSR-carrying sperm therefore develop into males instead of females. Interestingly, PSR somehow avoids self-elimination and successfully segregates with the maternal chromosomes and is therefore transmitted to the next generation. Because of this reproductive system, paternal genome elimination (PGE) induced by PSR converts female-destined embryos into PSR-carrying, and thus -transmitting, males (Fig. 1).

Two important questions have perplexed researchers regarding drive in this B chromosome-insect system. One is: How does PSR cause PGE? In most animals, much of the sperm’s DNA is packaged with sperm nuclear basic proteins (SNBPs), including protamines, during the late stages of spermatogenesis (Das 1964; Braun 2001; Rathke et al. 2010; Kanippayoor et al. 2013). In a process known as the histone-to-protamine transition, histones are removed from the sperm’s DNA and they are replaced with protamines with the help of transition proteins (Loppin et al. 2015). The packaging of DNA with protamines facilitates the compartmentalization of the sperm’s genome into a state that is denser than histone-based chromatin for protection from oxidative damage and so that it fits into a smaller sperm nuclear volume (Eirín-López et al. 2006; Barati et al. 2020). Immediately after fertilization, when the sperm nucleus enters the egg cytoplasm, the protamines are rapidly removed, and the sperm’s DNA is repackaged with histones. This histone repackaging event is needed for the sperm and egg chromatin sets to be compatible with one another so that they can synchronously undergo S-phase and enter into the first mitotic division (Loppin et al. 2001, 2015; Hundertmark et al. 2017). In addition, specific histone post-translational modifications (PTMs), marks of the epigenome, must be enzymatically placed in certain regions of the newly repackaged paternal chromatin, helping to establish distinct chromatin domains (reviewed in Hundertmark et al. 2017). Previous work showed that PSR alters a subset of histone PTMs on the sperm’s chromatin during epigenome establishment, and it was suggested that these alterations directly or indirectly cause PGE (Aldrich et al. 2017). Specifically, histone H3 di- and tri-methylated at Lysine 9 (H3K9me2,3), histone H3 mono-methylated at Lysine 27 (H3K27me1), and histone H4 mono-methylated at Lysine 20 (H4K20me1) become abnormally enriched across the paternal chromatin during this time (Aldrich et al. 2017; Aldrich and Ferree 2017). In contrast, other examined histone PTMs normally appeared in distinct regions in the presence of PSR, thus demonstrating that the effect is restricted to certain aspects of chromatin (Aldrich et al. 2017; Aldrich and Ferree 2017). An outstanding question is how PSR causes these chromatin alterations. A more recent study identified a PSR-expressed gene, named *haploidizer*, which is required for PGE; reduction of *haploidizer* transcripts in the testis by systemic RNA interference (sRNAi) caused suppression of the histone PTM abnormalities, PGE, and male-biased sex ratio distortion (Dalla Benetta et al. 2020), thereby implicating this gene as an effector of chromatin alteration and PGE. However, it is currently not understood mechanistically how *haploidizer* functions in this process.

A second, less examined question is: How does PSR evade its own chromatin-altering activity? This question is particularly compelling given that PSR resides within the same nucleus as the sperm-derived chromatin during spermatogenesis and until the first chromatin abnormalities appear just after fertilization (Swim et al. 2012; Aldrich et al. 2017). Thus, PSR is likely to be exposed to its own PGE-inducing activity within this developmental time frame. Interestingly, PSR was found to be visibly devoid of H3K27me1 and H4K20me1 when these histone PTMs become abnormal on the paternal chromatin (Swim et al. 2012; Aldrich et al. 2017). Given that PSR does not undergo self-elimination, it is not unexpected that the B chromosome would be devoid of these histone PTMs if they play a role in PGE. However, these observations beg the question: how can PSR behave so differently from the normal chromosomes in this capacity?

In this study, we investigated several hypotheses pertaining to PSR’s ability to evade self-elimination. One hypothesis is that PSR may evade chromatin alteration by its own PGE activity by not participating in the histone-to-protamine transition. Notably, in some examined organisms, certain chromosomal sub-regions, including the centromeres, and even whole chromosomes are known to retain histones during sperm formation (Raychaudhuri et al. 2012; Rathke et al. 2014; Torres-Flores and Hernández-Hernández 2020). Thus, these chromosomes and chromosomal sub-regions do not undergo the histone-to-protamine transition or de novo histone PTM placement following fertilization. If the same were true of PSR, then it would have a means of escaping chromatin disruption and self-elimination.

Alternatively, PSR may avoid genome elimination because of a unique DNA sequence or chromatin property that makes it naturally recalcitrant to its own chromatin-altering activity. Such a property may be specific to PSR’s activity, or, more broadly, it may include immunity to other PGE-inducing heritable agents, such as the intracellular bacterial symbiont, *Wolbachia*. Known to infect many insect species worldwide (Hilgenboecker et al. 2008; Zug and Hammerstein 2012), certain strains of *Wolbachia* induce conditional male sterility known as cytoplasmic incompatibility (CI), in which bacterial infection in the testis results in PGE in a manner reminiscent of that caused by PSR (Reed and Werren 1995; Breeuwer and Werren 1990; Riparbelli et al. 2007; Callaini et al. 1997; Sullivan and O’Neill 2017). To cause PGE, CI-inducing *Wolbachia* alters the sperm’s chromatin so that it fails to resolve into chromosomes (Fig. 1). In diploid insects like *Drosophila melanogaster*, this effect causes death, whereas in *N. vitripennis*, fertilized embryos develop as haploid males (Tram et al. 2006). However, if the egg is infected with the same *Wolbachia* strain—i.e., a condition known as rescue—PGE is suppressed (Werren 1997; Zabalou et al. 2004; Shropshire et al. 2018). These activities selectively favor *Wolbachia*-infected female hosts, ultimately leading to enhanced bacterial transmission because *Wolbachia* is maternally transmitted (Caspari and Watson 1959; Turelli 1994; Werren et al. 2008). A previous study showed that PSR typically does not survive the CI effect of *Wolbachia*, although some PSR chromosomes undergo large terminal deletions and are transmitted to the embryo (Beukeboom and Werren 1993), suggesting that PSR may be only partially sensitive to *Wolbachia*’s CI activity. However, little is known about the impact of *Wolbachia* on PSR at the microscopic and molecular levels. Investigating these possibilities will help to better understand the extreme drive behavior of PSR and, more broadly, how B chromosomes differ from the A chromosomes.

## Methods

### Maintenance of PSR and *Wolbachia*-infected lines

The PSR chromosome was maintained in the *AsymC* wasp line, which is wild type and has been cured of *Wolbachia* via antibiotic treatment (Werren et al. 2010). For propagation of the PSR+ line, virgin *AsymC* females were individually crossed with *AsymC* PSR+ males. Twenty-five of these pairwise crosses were set up during each generation. Crosses resulting in all-male broods were selected for further propagation. For the *Wolbachia* experiments, we used a wild-type line, *LbII*, which was originally collected in Leiden, The Netherlands. *LbII* is co-infected with the *N. vitripennis Wolbachia* A and B strains and is the line from which the *AsymC* line was derived (Breeuwer and Werren 1995). To perform the CI crosses, *AsymC* females (uninfected) were crossed with *LbII* males. For the rescue crosses, we used males and females from the *LbII* line. Husbandry of all wasp lines was conducted as previously described (Aldrich et al. 2017).

### Embryo collection, fixation, and staining

To collect wasp embryos for microscopic analysis, groups of 3–4 mated females were allowed to oviposit into host *Sarcophaga bullata* pupae for 0–1.5 h. The parasitized host pupae were then separated from the wasps. The embryos were removed from the host pupae with ultrafine forceps and carefully transferred into a 10 mL glass vial with a screw top. Embryos were immediately fixed in the following solution, adding the listed ingredients in this order: 3 mL heptane, 1.5 mL 1x Phosphate Buffered Saline (1× PBS), and 600 mL 37% formaldehyde. The vial containing embryos and fixative was gently agitated for 30 min. Subsequently, embryos were transferred onto a small piece of Whatman paper and allowed to dry for 30 s. The embryos were then transferred with gentle pressure onto double-sided adhesive tape pasted onto the bottom of a clean 22 mm plastic Petri plate. A small aliquot of 1× PBT (1× PBS with 0.1% Triton-X 100) was added to the immobilized embryos to hydrate them. The embryos were then devitellinized with either a 28- or 30-gauge hypodermic needle. The devitellinized embryos were transferred to a microfuge tube and washed three times with 1× PBT and either stored under refrigeration for up to 1 week or used immediately for immunostaining.

For immunostaining, formaldehyde-fixed embryos were incubated with diluted primary antibodies (see subsequent sections) overnight at 4 °C with gentle agitation. Subsequently, the embryos were washed three times with 1x PBT and stained with fluorescently conjugated secondary antibodies for 1 h in darkness. Secondary antibodies used were anti-rabbit Cy3 and anti-mouse Cy5 (1:300, Invitrogen, Thermo Fisher, Inc. USA). Embryos were washed three times in 1x PBT and either mounted on a slide with Vectashield mounting medium with DAPI (Vector Laboratories, Inc., USA) or used for DNA FISH before mounting. For DNA FISH, following antibody staining and washes, embryos were re-fixed in darkness for 45 min in 4% paraformaldehyde. Subsequently, the embryos were washed three times in 2× saline-sodium citrate and Tween-20. The remaining steps of the procedure were conducted exactly as described previously (Swim et al. 2012). Imaging was conducted using a Leica TCS SPE confocal microscope. Images were collected as either single images or as Z-series for each laser channel and subsequently merged. Images were exported in high-quality JPEG format and processed using Adobe Photoshop CSF v.12.

### Testing if PSR participates in the histone-to-protamine transition

To determine if PSR undergoes the histone-to-protamine transition, systemic RNA interference (sRNAi) was used to post-transcriptionally target the expression of *hira*, a gene whose encoded protein is required for nucleosome assembly following protamine removal in the egg’s cytoplasm (Bonnefoy et al. 2007). sRNAi knockdown of *hira* was performed by microinjecting double-stranded RNA (dsRNA) that is complementary to the *hira* transcripts into female pupae (Lynch and Desplan 2006; Dalla Benetta et al. 2020). To generate dsRNA, PCR was used to amplify a 753 bp region of *hira* that contained a T7 promoter at the 5′ and 3′ ends. The primers used to amplify this fragment were 5’-AAT ACG ACT CAC TAT AGG GGG TGG GTC AAC TGC TCA AAT-3’ (forward) and 5’-TAA TAC GAC TCA CTA TAG GGA CGT TTG GCT GAA CGA TTT C-3’ (reverse). This fragment was bidirectionally transcribed overnight at 37 °C using the Megascript RNAi Kit (Ambion, Austin, Texas, USA). Exonuclease digestion was conducted to remove DNA and single-stranded RNA, and the dsRNA was subsequently purified according to the kit’s protocol. Last, dsRNAs were precipitated with ethanol, redissolved in water, and stored at −80 °C. *N. vitripennis* female pupae were injected with 4 μg/μL dsRNA that was mixed with a light dilution of red food coloring following a previously described procedure (Lynch and Desplan 2006; Dalla Benetta et al. 2020). dsRNA injections were performed under continuous injection flow with the Femtojet 4i microinjector (Eppendorf) using microneedles made with aluminosilicate glass filaments and a Sutter needle-pulling instrument. Pupae were injected into the ventral side of the posterior abdomen. Injections were confirmed by monitoring a pink coloration in the abdomen. Injected wasp pupae were incubated at 25 °C on an agar/1× PBS Petri dish. After emergence, each female was mated individually either with a single wild type or PSR+ male (Dalla Benetta et al. 2020). Resulting embryos were stained with either rabbit anti-H3K9me2,3 (Active Motif) or rabbit anti-pan core histones (Active Motif), and the PSR chromosome was painted using DNA FISH as described above with the following PSR-specific ssDNA probe: 5’-CAC TGA AAA CCA GAG CAG CAG TTG AGA-3’. This sequence was synthesized by IDT Inc. (USA) and the 5’ end was conjugated with Alexa-488. Microscopic analysis of embryos was conducted as described above.

To assess the efficiency of sRNAi knockdown, total RNA was extracted from ~20 ovaries from untreated and RNAi-treated females. RNA extractions were performed using TRIzol reagent according to the manufacturer’s instructions. Each RNA sample was subjected to deoxyribonuclease treatment to eliminate any contaminating DNA. Approximately 1 μg of total RNA was reverse transcribed with oligo-dT and hexamer primers (1:6 ratio) with the RevertAid H Minus First Strand cDNA Synthesis Kit (Fermentas, Hanover, MD, USA). The cDNA was then diluted 30-fold. qPCR was performed with SYBR Green (Genesee Scientific). Diluted cDNA (4 μL) was used for each 20 μL reaction containing primers at a final concentration of 0.2 μM and 10 μL of SYBR Green buffer solution. The primer sequences for *hira* qPCR were: 5’-GGA GAA TCA CGC CAA TGT TT-3’ (forward) and 5’-GAA CGA TTT CAT CGC GTT TT-3’ (reverse). Three technical replicates for each reaction were performed to correct for experimental error. Two genes (*ef1a* and *ak3*) were used as references for the normalization of the data after the confirmation that their expression levels were constant between tissues (Dalla Benetta et al. 2019). The primer sequences for these genes were: 5’-CAC TTG ATC TAC AAA TGC GGT G-3’ (*ef1a* forward); 5’- CCT TCA GTT TGT CCA AGA CC-3’ (*ef1a* reverse); 5’-AAT TCA ATC GGG TTC TGC TC-3’ (*ak3* forward) and 5’- CAG CAT CTC ATC TAA CTT CTC TG-3’ (*ak3* reverse). Relative expression levels of *hira* to the reference genes were calculated by normalizing the expression data with LightCycler 96 software.

### Assessing how *Wolbachia* affects the segregation of PSR

To determine to what degree PSR is sensitive to the chromatin-altering activity of *Wolbachia*, the mitotic behavior of PSR was visualized in young CI embryos produced from crosses of PSR+ *Lbll* males to *AsymC* (uninfected) females, which are cytoplasmically incompatible and therefore should result in the destruction of the paternal chromatin and PSR. The PSR+ *Lbll* males were produced by crossing PSR+ *AsymC* males with *Lbll* females. The PSR chromosome was painted using DNA FISH as described above.

### Examination of *Wolbachia*-induced chromatin alterations to the PCM and to PSR

CI embryos were microscopically examined to determine how *Wolbachia* affects the sperm-derived chromatin during the first embryonic mitotic division. To perform the CI crosses, *AsymC* females were crossed with *LbII* males. For the rescue crosses, we used males and females from the *LbII* line. Young embryos were collected, fixed, and antibody-stained as described above. Emphasis was placed on histone PTMs known to be altered by PSR (Aldrich et al. 2017). The primary antibodies were: rabbit anti-H3K9me2,3 (Active Motif), rabbit anti-H3K27me1 (Active Motif), rabbit anti-H4K20me1 (Active Motif), and rabbit anti-panH4ac (Millipore). All primary antibodies were used at a dilution of 1:500 in 1x PBT. PSR+ embryos were also prepared and stained for comparison, and DNA FISH was used to paint PSR. The PSR chromosome was examined under high magnification using confocal microscopy.

### Effect of PSR on the ribosomal DNA locus

The chromatin state of the ribosomal DNA (rDNA) locus was examined in the presence of PSR by using antibody staining to detect H3K9me2,3 and H4K20me1 followed by DNA FISH as described above. rDNA was visualized using a combination of two probes that are complementary to different regions of the *N. vitripennis* 18S Intergenic Spacer Repeat repeat: 5′-TTA GAC TTT TTC GAG CCT CCG AGA-3′ and 5′-ATT GAC GCT CGC ACA TCA CTC ATT-3. Both probes were conjugated at the 5’ end with Cy5 (IDT).

## Results

### Testing if PSR participates in the histone-to-protamine transition

**Fig. 2:**
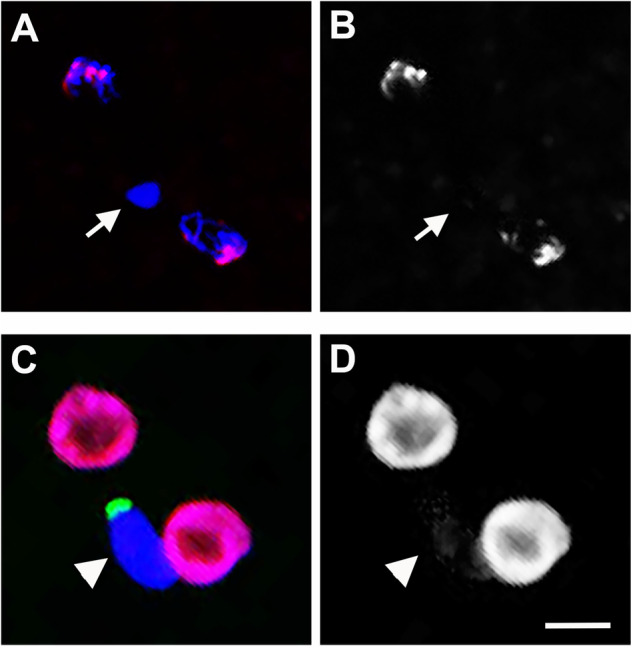
The effect of hira RNAi targeting on PSR. **A** A PCM (white arrow) is formed in a wild-type embryo from sRNAi targeting of *hira*. DNA is blue and H3K9me2,3 is red. **B** The H3K9me3 signal from (**A**) is shown in gray. There is no visible signal in the PCM. **C** An RNAi-treated embryo containing PSR (green). Signal from an antibody that recognizes all core histones is red. DNA is blue. **D** The histone signal from (**C**) is shown separately in gray for contrast. There are only trace amounts of histones on the PCM (white arrowhead). Scale bar is 5 um.

It is possible that PSR may evade self-elimination by not participating in the histone-to-protamine transition. If true, PSR would be exempt from histone repackaging and establishment of the epigenome following fertilization, thus being spared of abnormal histone PTM alterations. To test this hypothesis, sRNAi was used to post-transcriptionally target the expression of *hira*, which is required for the placement of H3.3 following protamine removal in the egg cytoplasm. H3.3-based nucleosomes appear on the paternal chromatin in a replication-independent manner before replacement with H3-based nucleosomes (Loppin et al. 2015). sRNAi targeting of *hira* in wild-type *N. vitripennis* females, which had an efficiency of 92% transcript reduction (Supplementary Table 1), caused the formation of a PCM in fertilized embryos (Fig. 1; *N* = 9/9 embryos). The egg-derived chromatin in the wasp was not affected by *hira* targeting (Fig. 2). In addition, whereas the egg-derived chromatin showed normal levels of H3K9me2,3 in its pericentromeric regions, this modification was visibly absent from the sperm-derived chromatin (Fig. 2A, B). We saw no other cellular abnormalities, suggesting that the sRNAi effect is specific to the paternal chromatin. When allowed to develop, these fertilized embryos developed into all-male broods (*N* = 23/24 broods from individual pairwise crosses), suggesting a complete loss of the sperm-derived chromatin and female-to-male conversion due to the sRNAi treatment. These results demonstrate a clear, expected set of phenotypes resulting from sRNAi targeting of *hira* in *N. vitripennis*, and that *hira* is required for processing of the sperm-derived chromatin in this insect.

Next, embryos produced from crosses between *hira*-sRNAi-treated females and PSR-carrying males were examined. In these embryos, PSR was completely blocked from segregating away from the PCM (Fig. 2C, D; *N* = 6/6 embryos). To be certain of the sRNAi effect on the sperm-derived chromatin, these embryos were stained with an antibody that highlights core histones. Only trace amounts of histones were visible on the paternal set, in contrast to control PSR-carrying embryos (i.e., those from untreated mothers), which are known to contain high histone levels (Fig. 2C, D) (Swim et al. 2012; Aldrich et al. 2017). Thus, PSR, like the essential chromosomes, requires the activity of *hira* to segregate from the PCM.

### Assessing how *Wolbachia* affects the segregation of PSR

CI embryos were first examined to confirm the PGE activity of *Wolbachia*. *A* PCM formed in all uninfected eggs fertilized by sperm from *Wolbachia-*infected males (*N* = 49). The PCM caused by *Wolbachia* is considerably more diffuse and less densely stained than the PCM caused by PSR (Supplementary Fig. 1).

**Fig. 3:**
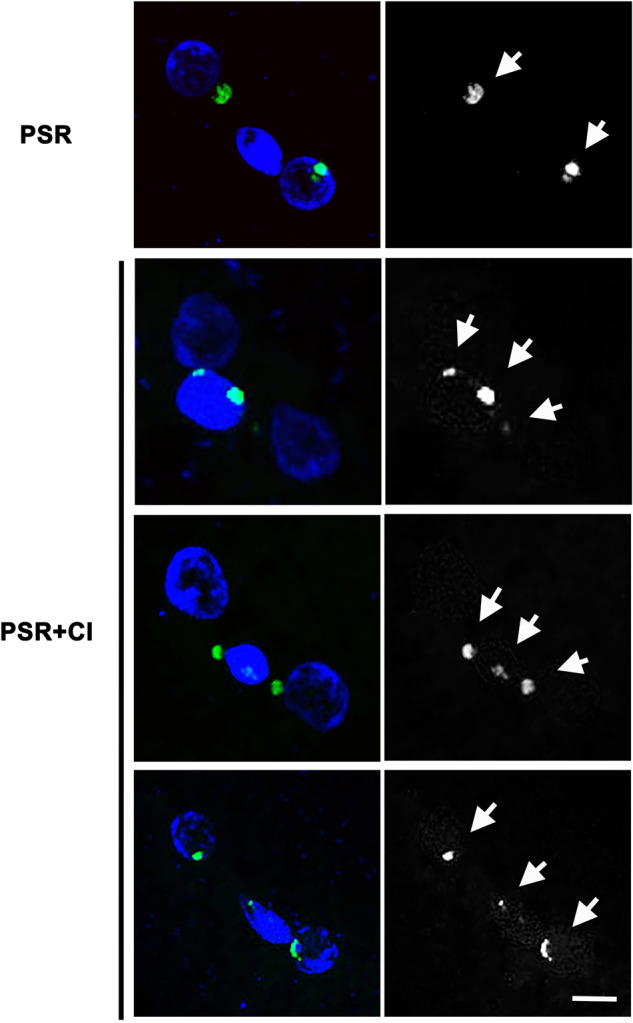
*Wolbachia* partially affects the ability of PSR to segregate from the PCM. DNA is blue and PSR is green (first column) and gray (second column). White arrows indicate PSR chromatids and fragments. Scale bar is 5 um.

Embryos were then examined to determine how CI affects the segregation behavior of PSR during the first mitotic division. In the absence of *Wolbachia*, PSR successfully segregated away from the PCM in nearly all examined embryos (*N* = 34/36) (Fig. 3). In these embryos, each of PSR’s sister chromatids associated with a daughter nucleus derived from the egg’s hereditary material (Fig. 3). In two embryos, one of the two sister PSR chromatids remained in the PCM (not shown). However, in embryos from *Wolbachia*-infected, PSR+ fathers, PSR exhibited a range of phenotypes regarding its segregation. In some embryos (*N* = 11/33), the sister PSR chromatids separated from one another but did not leave the chromatin mass (Fig. 3). In other embryos, the sister PSR chromatids separated from one another and either partially (*N* = 16/33) or fully (*N* = 6/33) escaped from the chromatin mass, in some cases joining the egg-derived nuclei (Fig. 3). In both cases, remnants of PSR were left behind in the PCM, showing that PSR becomes fragmented when it segregates. In all of these embryos, we never observed fragments outside the PCM that did not contain PSR-specific sequences.

### Examination of *Wolbachia*-induced chromatin alterations to the PCM and to PSR

CI embryos were examined to determine how *Wolbachia* affects the chromatin state of the PCM. Three specific histone PTMs—H3K9me2,3, H3K27me1, and H4K20me1—were scrutinized because they are known to be disrupted by PSR (Supplementary Fig. 2) (Aldrich et al. 2017). H3K27me1 was similarly disrupted in PSR+ embryos and in CI embryos; in both conditions, this histone PTM was abnormally heightened and distributed broadly across the PCM (Supplementary Fig. 2). However, in contrast to PSR+ embryos, H4K20me1 appeared to be only mildly disrupted and H3K9me2,3 was unperturbed on the PCM in CI embryos (Supplementary Fig. 2). The H3K27me1 and H4K20me1 disruptions and PGE were strongly suppressed by the presence of *Wolbachia* in the egg (Supplementary Fig. 2).

**Fig. 4:**
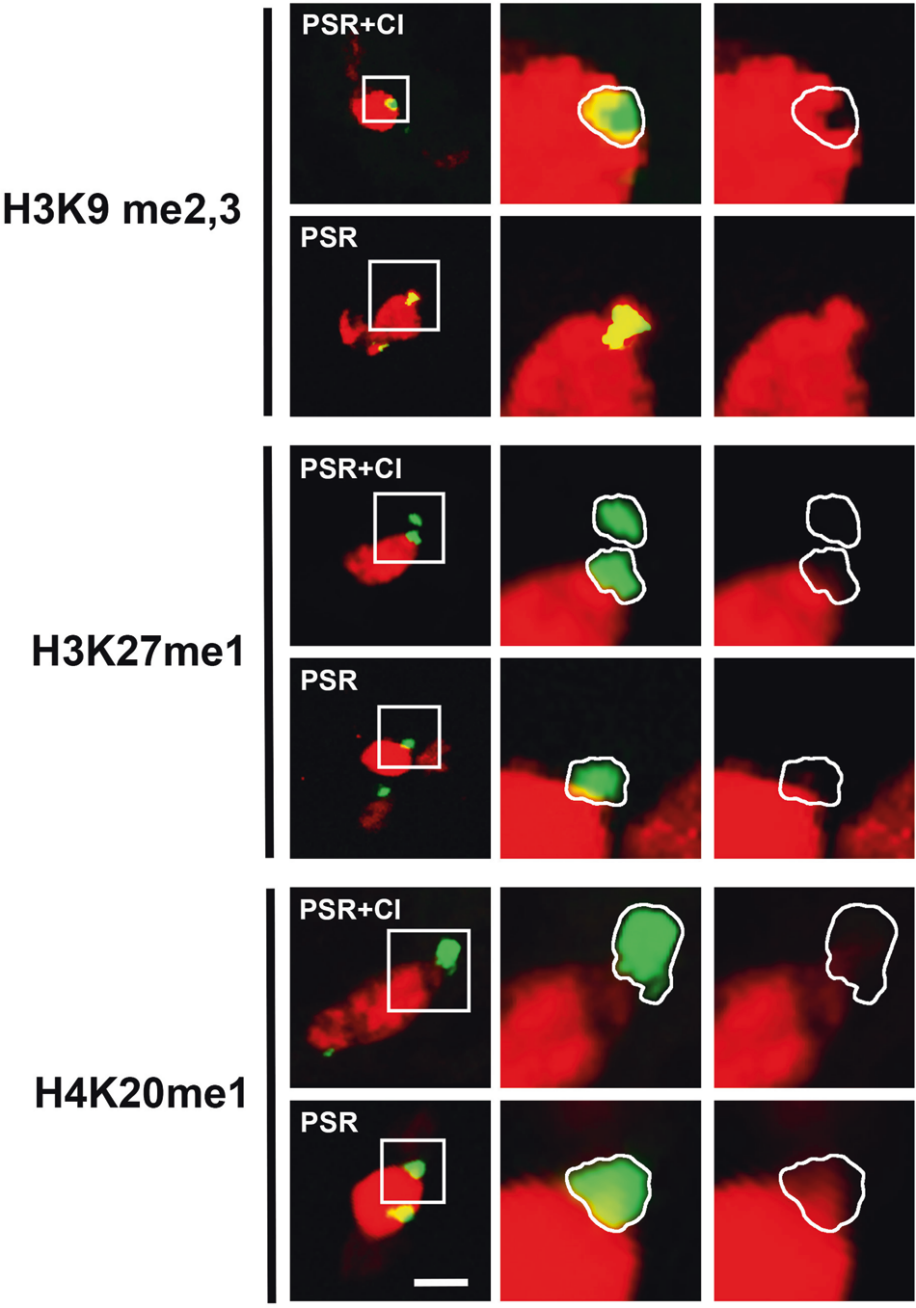
The effects of *Wolbachia* on the chromatin state of PSR. Each histone modification is shown in red and PSR is green. The images in the second and third columns are higher magnifications of the boxed regions shown in the first column. The PSR channel has been removed in the third column for clear visualization of the histone modifications. Scale bar is 5 um.

The PSR chromosome was then examined in CI embryos to determine if *Wolbachia* alters H3K27me1 and H4K20me1 on the B chromosome as it does on the PCM. Just as PSR is devoid of these histone PTMs in PSR+ embryos (Fig. 4) (Aldrich et al. 2017), it is also devoid of them in CI embryos (Fig. 4). H3K9me2,3, which is normally enriched on PSR presumably because it is heterochromatic in nature, was absent from the B chromosome in CI embryos (Fig. 4). Therefore, *Wolbachia* only affects one of these three histone PTMs on PSR.

### Effect of PSR on the rDNA locus

**Fig. 5:**
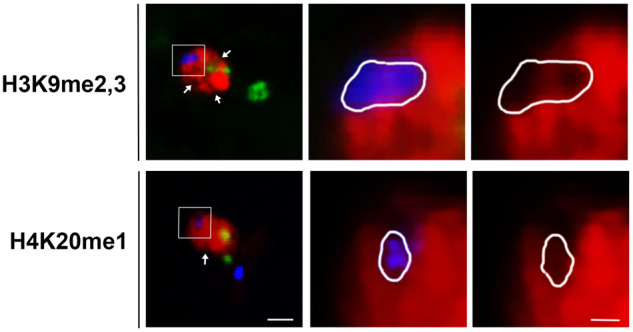
The chromatin of the rDNA locus and other A chromosomal regions are not altered by PSR. Images are from uninfected embryos. Two histone modifications, H3K9me2,3 and H4K20me1, are shown separately in red. The white arrows indicate regions of the PCM that are devoid of these marks. The boxed regions of the paternal chromatin are shown as higher magnifications in the second and third columns. rDNA (blue) is shown in the middle column but has been removed in the third column to demonstrate the lack of histone modifications. PSR is green. Scale bar in the low magnification image is 5 and 1 uM in the high magnification image.

In PSR+ embryos, while the majority of the PCM showed abnormal histone PTM patterns in the presence of PSR, there were regions near the nuclear periphery that appeared less enriched in these marks (Fig. 5). Given that pericentromeric heterochromatin is known to localize near the nuclear periphery (Holla et al. 2020; Bank and Gruenbaum 2011), it was hypothesized that these regions consist of heterochromatic sequences. To test this possibility, the rDNA locus, which is located within pericentromeric heterochromatin, was visualized in PSR+ embryos. Similar to PSR, the rDNA locus was devoid of H3K9me2,3 and H4K20me1 (Fig. 5). Thus, rDNA, and other unidentified regions likely to be heterochromatic, may be naturally resistant to the chromatin-altering activity of PSR.

## Discussion

Genome and chromosome elimination events are known to occur in several different eukaryotic species as a part of their normal development (Brown and Nur 1964; Berry 1939; Pigozzi and Solari 2005; Jaron et al. 2022). However, PGE in the jewel wasp is exceptional because it is caused by the non-essential B chromosome, PSR (Werren et al. 1987; Nur et al. 1988). The activity that causes PSR-induced PGE occurs either during spermatogenesis or in the egg cytoplasm immediately before the first embryonic mitotic division (Dalla Benetta et al. 2020). The fact that PSR does not undergo self-elimination despite being associated with the sperm-derived chromatin until that time underscores some remarkable, unknown characteristic(s) of this B chromosome that distinguish it from the A chromosomes.

We originally speculated that PSR might evade self-elimination by not participating in the histone-to-protamine transition, as is the case for certain chromosomal regions and whole chromosomes in other organisms (Raychaudhuri et al. 2012; Rathke et al. 2014). It was not possible to test this hypothesis by manipulating the wasp’s protamines because they have not yet been identified; efforts to bioinformatically identify the protamine gene(s) have been hindered presumably because SNBPs are known to evolve rapidly, obscuring signatures of homology (Retief et al. 1993; Kasinsky et al. 2011; Oliva and Dixon 1991; Wyckoff et al. 2000). However, as an alternative, we post-transcriptionally targeted the gene *hira*, whose encoded protein facilitates the addition of the transitional histone H3.3 to the paternal DNA following removal of protamines in the egg (Bonnefoy et al. 2007). Similar to the *hira*-sRNAi phenotype observed here in *N. vitripennis* (Fig. 2), a *hira* loss-of-function allele in *D. melanogaster* caused the failure of the paternal chromatin to decondense following entry into the egg (Bonnefoy et al. 2007). In this mutant background, the *D. melanogaster* protamines Prot-A and Prot-B properly disappeared from the paternal chromatin, showing that *hira* is not responsible for protamine removal in this organism (Bonnefoy et al. 2007). However, the *hira*-specific loading of H3.3 is required specifically for regions of the genome undergoing the histone-to-protamine transition. A logical prediction is that if PSR segregates properly in the jewel wasp when *hira* is targeted by RNAi then PSR likely is not packaged with protamines in the sperm and therefore does not need *hira* activity. Our finding that PSR becomes completely immobilized within the PCM when *hira* is sRNAi-targeted argues that the B chromosome does indeed undergo the histone-to-protamine transition. Thus, in this regard, PSR is not unlike the A chromosomes.

The use of *Wolbachia* in this study revealed comparative insights into how the PGE activities of this bacterium and PSR differ, as well as the capability of PSR to avoid them. Whereas PSR causes disruption of the H3K9me2,3, H3K27me1, and H4K20me1 histone PTMs on the PCM (Supplementary Fig. 2; Aldrich et al. 2017), *Wolbachia* alters H3K27me1 in a similar manner but it disrupts H4K20me1 only slightly and has no obvious effect on H3K9me2,3 (Supplementary Fig. 2). In addition, the PCM caused by *Wolbachia* appears less dense than the PCM caused by PSR, an observation that echoes those made in a previous study (Reed and Werren 1995). These observations, particularly the differences in histone PTM disruption, argue that *Wolbachia* and PSR induce PGE not through differences in strength of the same mechanism, but instead through different mechanisms. This claim is supported by the causal genes expressed by each agent. Recent work has identified two *Wolbachia*-phage genes, *cifA* and *cifB*, which, when transgenically expressed together, can recapitulate the CI and rescue phenotypes (Adams et al. 2021; Shropshire et al. 2018; LePage et al. 2017; Shropshire and Bordenstein 2019). In particular, *cifB*, when expressed in the *D. melanogaster* testis, is sufficient to cause PGE in a manner that mimics bacterial activity (Lindsey et al. 2018; Shropshire and Bordenstein 2019). Subsequently, it was shown that the *CifB* protein, containing a deubiquitylation domain, localizes within the sperm’s nucleus and is retained at DNA regions that exhibit replication stress in the young embryo (Horard et al. 2022). So far, only one PSR-expressed gene, *haploidizer*, has been identified as necessary for causing PGE in *N. vitripennis* (Dalla Benetta et al. 2020). This gene encodes a putative protein with a C4 zinc finger DNA-binding domain and no other recognizable domains (Dalla Benetta et al. 2020). Thus, no functional domain similarity exists between *haploidizer* and *Wolbachia*’s *cif* genes, implying different molecular functions.

An important question is to what degree CI affects PSR’s ability to segregate. The fact that segregating chromosomal fragments of PSR could be generated by CI in a previous genetic study (Beukeboom and Werren 1993) suggested that PSR is indeed affected by *Wolbachia* but that the effect is incomplete. Microscopic analysis performed here confirmed this idea, showing that while in some embryos one or both PSR chromatids remained inside the PCM, in other embryos large fragments of PSR moved outside the PCM, occasionally associating with the egg-derived nuclei. In all cases, small remnants of PSR’s DNA remained in the PCM. The success of PSR to partially segregate in a CI background makes sense in light of the finding that the B chromosome itself is devoid of visible H3K27me1 and H4K20me1, two histone PTMs that the bacterium disrupts on the PCM. If these two histone PTMs are important for chromatin disruption by *Wolbachia*, then their absence on PSR is consistent with its ability to partially escape the PCM. Interestingly, although PSR is heterochromatic, it is abnormally devoid of H3K9me2,3 in the presence of *Wolbachia*. This particular pattern may indicate a specific alteration of heterochromatin that incompletely blocks the segregation of PSR. This idea is supported by experiments in human fibroblasts in which chemical manipulation of histone deacetylation led to a depletion of the heterochromatin protein 1 on mitotic chromosomes (HP1) (Cimini et al. 2003). This effect hindered sister chromatid separation along the chromatid arms, resulting in chromosome bridges between daughter nuclei (Cimini et al. 2003). In addition, *Wolbachia* was previously observed to cause chromosome bridges of the paternal chromatin during the later cleavage divisions in *N. vitripennis* (Reed and Werren 1995). In the case of PSR, its diminutive arm length may result in more successful sister chromatid separation and departure from the PCM, and the remaining fragments may result from chromosome bridge breaks.

What properties might explain the ability of PSR to partially escape the PGE activity of *Wolbachia* and fully evade its own activity? It is possible that PSR has a chromatin composition that differs from that of the A chromosomes. A previous study showed that over 90% of PSR’s sequences are unique, not being found in the wasp’s genome (Dalla Benetta et al. 2020). Approximately 70% of these sequences belong to a family of complex satellite DNA repeats (Eickbush et al. 1992; Dalla Benetta et al. 2019, 2020). Interestingly, these repeats contain a highly conserved palindromic heptamer motif that is similar to those recognized by certain transcription factors and other DNA-binding proteins (Eickbush et al. 1992). Indeed, it is known that in *D. melanogaster*, certain satellite DNA repeats can associate with specific DNA-binding factors, giving some of these sequences unique chromatin properties (Gaff et al. 1994; Raff et al. 1994; Torok 2000). Perhaps due to a similar association with specific chromatin factors, PSR’s sequence composition may somehow make the chromatin of this B chromosome resistant to its own activity that causes PGE.

An alternative possibility is suggested by the observation that not all of the paternal chromatin shows signs of disruption by PSR. In particular, certain regions of the A chromosomes located around the periphery of the PCM are, like PSR, devoid of the abnormal histone PTMs, H3K9me2,3 and H4K20me1. One of these regions was identified here as the rDNA locus (Fig. 5), which is located within pericentromeric heterochromatin. The identities of the other unmarked regions remain to be identified. However, given their positions near the nuclear periphery, it is likely that they also are heterochromatic. Thus, it is possible that the PGE activity of PSR targets some aspect of euchromatin, and because PSR lacks euchromatic arms, it would not be affected. In addition, while the pericentromeric heterochromatin of the A chromosomes also would not be affected by PSR’s activity, they would nevertheless fail to segregate because of their large euchromatic content. Testing these ideas will benefit from a functional understanding of the *haploidizer* gene and its relationship to chromatin.

## Supplementary information

Supplementary material figures

Supplementary Table 1

## Data Availability

The data have been submitted to Dryad and can be accessed at 10.5061/dryad.p5hqbzkv0.
