# Supplementary material for: Ability of a selfish B chromosome to evade genome elimination in the jewel wasp, Nasonia vitripennis
Source: Heredity (Edinb). 2023 Jul 31;131(3):230–7. doi: 10.1038/s41437-023-00639-0 (PMC10462710; doi:10.1038/s41437-023-00639-0)
Supplement: Supplementary file 1 — Supplementary material figures [file 41437_2023_639_MOESM1_ESM.pdf]

SUPPLEMENTARY FIGURES AND LEGENDS

Supplementary Figure 1

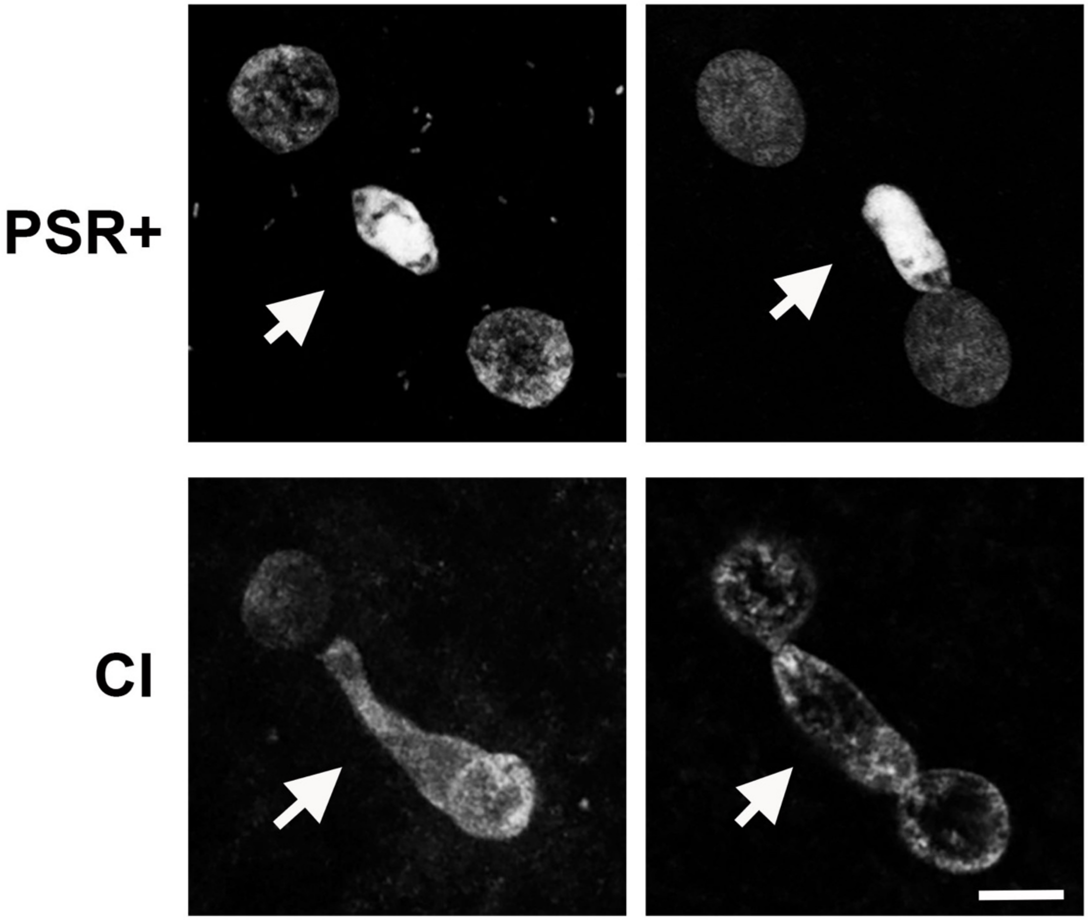

Supplementary Figure 2

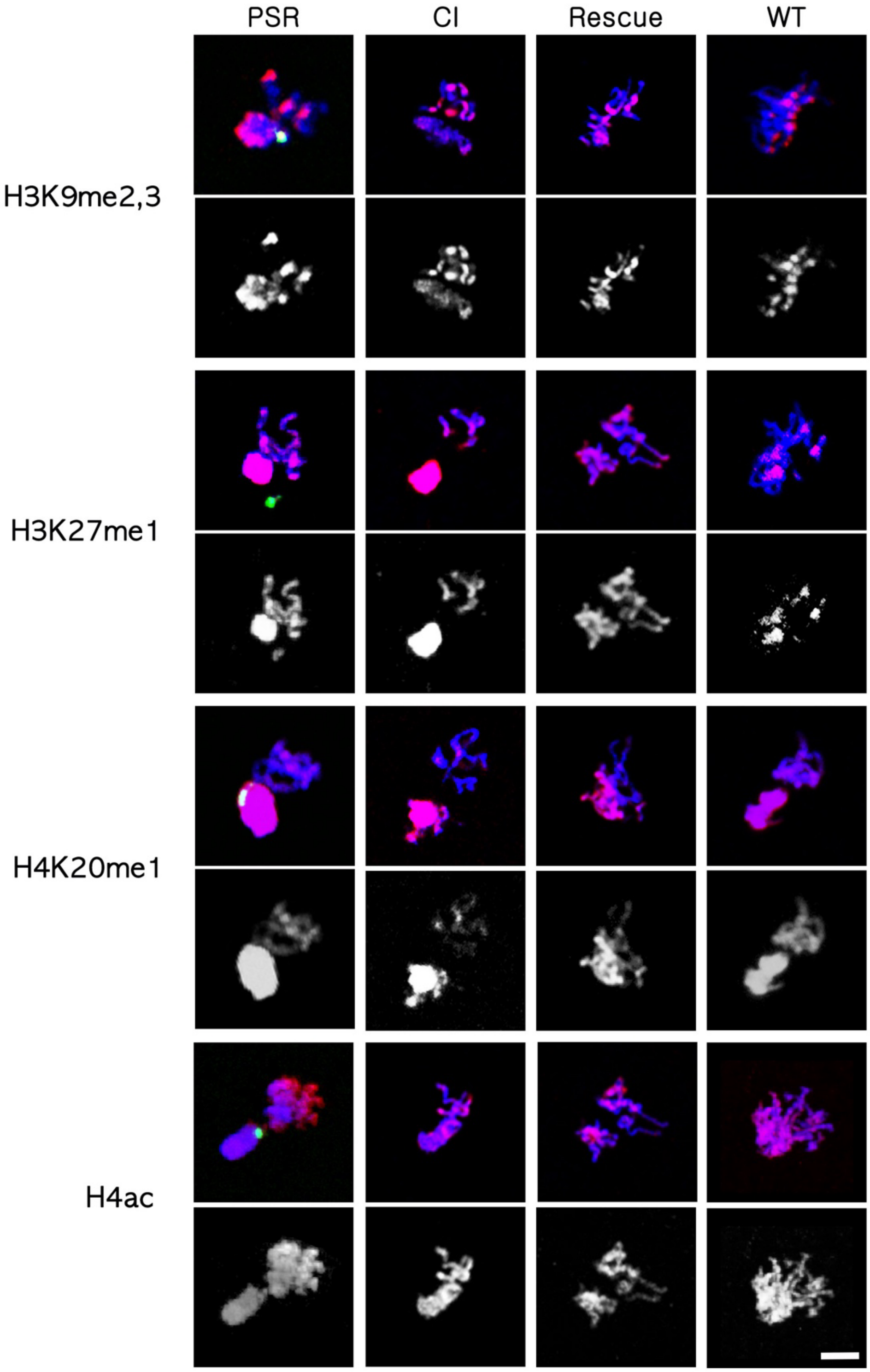

Supplementary Figure 1. *The PCMs caused by PSR and Wolbachia have different appearances*. PCM caused by PSR (A and B) and *Wolbachia* (C and D) during the first embryonic mitotic division in the young embryo. The PCM is associated with (A) neither daughter nucleus, (B and C) one daughter nucleus, or (D) both daughter nuclei. DNA is shown in grey. Scale bar is 5 um.

Supplementary Figure 2. *Histone modifications that are altered on the PCM by PSR and Wolbachia* immediately after fertilization. Four different conditions are shown: PSR+ embryos (first column), CI embryos formed from *Wolbachia*-infected fathers and uninfected mothers (second column), rescue embryos formed from both *Wolbachia*-infected parents (third column), and embryos from wild type (WT) parents. Each panel depicts the paternal and maternal nuclei at metaphase of the first embryonic mitosis. For each histone modification, the top row shows the modification in red and DNA in grey, while the second row shows the modification in grey for maximum contrast. In the first column, PSR is green. DNA is blue in color panels. Scale bar is 5 um.
